# Pericardial Diseases in Cardiovascular Medicine: Contemporary Diagnosis, Risk Stratification, Multimodality Imaging, and Targeted Anti-Inflammatory Therapy

**DOI:** 10.31083/RCM50417

**Published:** 2026-06-26

**Authors:** Bum Sung Kim, Ji-won Hwang

**Affiliations:** ^1^Division of Cardiology, Department of Medicine, Konkuk University Medical Center, 05030 Seoul, Republic of Korea; ^2^Division of Cardiology, Department of Medicine, Ilsan Paik Hospital, Inje University School of Medicine, 10380 Goyang, Gyeonggi-do, Republic of Korea

**Keywords:** pericarditis, constrictive pericarditis, multimodality imaging, echocardiography, magnetic resonance imaging, interleukin-1

## Abstract

Pericardial diseases comprise a heterogeneous spectrum, ranging from self-limited acute pericarditis to recurrent inflammatory syndromes, large pericardial effusions, cardiac tamponade, and chronic constrictive pericarditis. Although acute pericarditis is often considered a benign condition, a substantial proportion of patients develop recurrent or persistent disease with prolonged symptoms and an impaired quality of life. Diagnostic uncertainty and suboptimal early management, particularly excessive corticosteroid use, may further contribute to the development of chronic disease and treatment resistance. Recent advances in cardiovascular imaging and anti-inflammatory therapy have substantially transformed the management of pericardial diseases. Multimodality imaging, including echocardiography, cardiac computed tomography (CT), and cardiac magnetic resonance (CMR) imaging, plays a central role in anatomical assessment, evaluation of hemodynamic impact, and differentiation of active inflammation from irreversible fibrotic remodeling. Meanwhile, an improved understanding of the autoinflammatory pathways involved in recurrent pericarditis has led to the development of targeted therapies, most notably interleukin -1 (IL-1) inhibitors, which have demonstrated robust efficacy and meaningful steroid-sparing effects in refractory disease. This review summarizes contemporary diagnostic and therapeutic strategies for pericardial diseases, with an emphasis on recurrent pericarditis and imaging-guided management. We propose a stepwise, precision-based approach that integrates clinical risk stratification, multimodality imaging, and targeted anti-inflammatory therapy to optimize patient outcomes.

## 1. Introduction

Pericardial diseases represent a fundamental, yet evolving, domain of cardiovascular medicine. Acute pericarditis is a common cause of nonischemic chest pain in both emergency and outpatient settings and has long been regarded as a benign, self-limiting condition. However, accumulating evidence has demonstrated that pericardial diseases comprise a broad spectrum of inflammatory and structural disorders characterized by heterogeneous clinical courses, prognoses, and therapeutic challenges [1,2,3,4,5]. In particular, recurrent and chronic constrictive pericarditis is currently recognized as a clinically significant condition associated with substantial morbidity, impaired quality of life, and increased healthcare needs [6,7,8].

Historically, the management of patients with pericardial disease has relied on a simplified framework based on the clinical presentation, electrocardiography findings, and the echocardiographic assessment of pericardial effusion, with empirical treatment centered on nonsteroidal anti-inflammatory drugs (NSAIDs) and early corticosteroid use [9,10]. Although effective for acute symptom relief, this approach fails to address disease heterogeneity, underlying inflammatory mechanisms, and long-term outcomes, leading to the development of recurrent inflammation, corticosteroid dependence, or chronic pericardial remodeling [6,11].

Contemporary concepts now frame pericardial disease as a dynamic continuum rather than as an isolated clinical entity. Acute pericarditis may progress to recurrent disease through persistent or relapsing inflammation, and in selected cases, the ongoing inflammatory activity may culminate in constrictive physiology [6,7,8,12]. Recurrent pericarditis is increasingly understood as an autoinflammatory condition driven by dysregulated innate immune responses, representing a paradigm shift with major diagnostic and therapeutic implications [13,14,15,16].

Despite advances in the understanding of immunopathological mechanisms, cardiovascular imaging has evolved rapidly. Multimodality imaging, including echocardiography, cardiac computed tomography (CT), ^18^F-fluorodeoxyglucose positron emission tomography (FDG-PET), and cardiac magnetic resonance (CMR) imaging, has become central to contemporary pericardial disease management, enabling the assessment of pericardial anatomy, hemodynamics, and inflammatory activity [3,17,18]. In particular, cardiac CMR imaging plays a key role in distinguishing active and potentially reversible inflammation from established fibrotic disease, which has important implications for therapeutic decisions and prognostic evaluation [17,18,19,20].

Moreover, the emergence of targeted anti-inflammatory therapies has transformed the treatment landscape, particularly for recurrent and refractory pericarditis. Interleukin -1 (IL-1) inhibition has demonstrated robust efficacy and meaningful steroid-sparing effects, addressing a long-standing unmet clinical need and prompting important updates to clinical guidelines emphasizing stepwise personalized treatment strategies [14,15,16,21].

Against this background, this review aims to synthesize the current evidence on the epidemiology, pathophysiology, diagnosis, and management of pericardial diseases, with a particular focus on patients with recurrent pericarditis, imaging-guided phenotyping, and targeted anti-inflammatory therapy [3,4,5,10,14]. By integrating advances in multimodal imaging and immunomodulatory treatment, we propose a practical precision-based framework for managing patients across the full spectrum of pericardial diseases.

## 2. Epidemiology and Etiology of Pericardial Diseases

### 2.1 Epidemiology

Acute pericarditis is the most common clinical presentation of pericardial disease and a frequent cause of nonischemic chest pain in routine practice. Epidemiological studies have suggested that acute pericarditis accounts for approximately 5% of emergency department visits for non–acute coronary syndrome chest pain and up to 1% of hospital admissions. These estimates likely underestimate the true incidence, as many mild cases are managed in outpatient settings or remain undiagnosed [1,9,10].

Although the short-term prognosis of acute pericarditis is generally favorable, recurrence is common, occurring in approximately 15–30% of patients after the first episode, and increases to nearly 50% among those with prior recurrence. Recurrent pericarditis is a major cause of morbidity, often resulting in repeated hospitalization, prolonged anti-inflammatory treatment, and impaired quality of life. Importantly, recurrence risk is influenced not only by disease severity but also by early treatment strategies, particularly an inappropriate or premature use of corticosteroids [6,9,13,14].

The epidemiology of pericardial disease varies substantially according to geographic and socioeconomic contexts. Idiopathic or presumed viral pericarditis is predominant in high-income countries. In contrast, infectious causes, most notably tuberculosis, remain the leading etiologies of pericardial effusion and constrictive pericarditis in low- and middle-income regions. Tuberculous pericarditis is associated with higher rates of chronic constriction and worse outcomes, highlighting the importance of regional epidemiology in clinical assessment [10,22,23,24]. Furthermore, noninfectious etiologies have become increasingly prevalent over time. Post-cardiac injury syndromes, autoimmune diseases, malignancy-related pericardial involvement, and iatrogenic causes are more frequently encountered, particularly in aging populations and in patients exposed to cardiac procedures, radiation therapy, or modern oncological treatments. These trends underscore the evolving epidemiology of pericardial disease and the need for a systematic diagnostic approach [8,10,12,22].

### 2.2 Etiology of Pericardial Diseases

Pericardial diseases have a broad and often overlapping range of responsible etiological mechanisms. Etiological classification is central to the diagnostic evaluation, risk stratification, and therapeutic decision-making [1,10,25].

Idiopathic and viral pericarditis are the most common etiologies of pericarditis in developed countries. Although viral triggers are frequently identified, direct viral identification is uncommon in routine clinical practice. Most cases follow a benign course; however, a subset progresses to recurrent disease driven by persistent immune activation rather than an ongoing infection. This distinction has important therapeutic implications [9,10,13].

Autoimmune and autoinflammatory pericarditis are associated with systemic inflammatory diseases, including systemic lupus erythematosus, rheumatoid arthritis, systemic sclerosis, and isolated autoinflammatory syndromes. Pericardial involvement may occur in parallel with systemic disease activity or may occur as an isolated manifestation. Notably, recurrent pericarditis without an identifiable systemic autoimmune disorder is increasingly recognized as an autoinflammatory condition mediated by dysregulated innate immunity, with IL-1 playing a central pathogenic role [11,13,14,16].

Postcardiac injury syndromes include pericarditis following myocardial infarction, cardiac surgery, percutaneous coronary intervention, or chest trauma. These immune-mediated syndromes typically develop days to weeks after injury. Although many patients respond to conventional anti-inflammatory therapy, recurrence may occur, particularly when treatment is inadequate or prematurely discontinued [10,26].

Malignancy-related pericardial disease results from direct tumor involvement, lymphatic obstruction, or treatment-related injury. Pericardial effusion is a common presentation and may be the initial manifestation of the underlying cancer. Malignant pericardial involvement carries important prognostic implications and often requires pericardiocentesis for diagnostic and therapeutic purposes [10,27,28].

Drug-induced pericardial disease is increasingly recognized in contemporary practice. Chemotherapeutic agents, immune checkpoint inhibitors, and other immunomodulatory agents have also been administered. This etiology should be taken into consideration when symptom onset is temporally related to medication exposure and alternative causes are not evident [10,29].

## 3. Anatomy and Pathophysiology of the Pericardium

### 3.1 Normal Pericardial Physiology

The pericardium is a fibroserous sac that surrounds the heart and proximal great vessels. It consists of an outer fibrous layer and an inner serous membrane with parietal and visceral components. Under normal conditions, a small amount of pericardial fluid reduces friction during cardiac motion and supports efficient ventricular filling [1].

The pericardium plays an important role in cardiac physiology. It regulates ventricular interdependence by constraining total cardiac volume. As a result, changes in the filling of one ventricle directly influence the other, ensuring coordinated biventricular function, particularly during respiration [30,31]. In addition, the pericardium limits excessive acute chamber dilation during sudden increases in venous return, thereby preventing abrupt increases in wall stress [30].

These physiological properties are clinically important when the pericardial compliance is reduced. Inflammation, fibrosis, or calcification restricts normal pericardial motion and impairs diastolic filling, leading to characteristic hemodynamic consequences [17].

### 3.2 Inflammatory Cascade in Pericardial Disease

Pericardial inflammation can be triggered by diverse insults, including viral infection, immune-mediated injury, cardiac interventions, malignancy, and drug-related toxicity. Despite the diverse etiologies, these conditions converge on a common pathway involving activation of the innate immune system and downstream cytokine signaling [22].

IL-1 is a key mediator of this inflammatory response. IL-1α and IL-1β promote endothelial activation, increase vascular permeability, and recruit inflammatory cells toward the pericardial space, leading to pericardial edema, effusion, and chest pain [13,32].

Dysregulation of the innate immune system plays a major role in recurrent pericarditis. Unlike classic autoimmune diseases, this condition typically lacks high-titer autoantibodies or antigen-specific T-cell activation. This autoinflammatory phenotype is responsible for the limited efficacy of conventional immunosuppressive therapies in some patients and provides a mechanistic basis for targeted cytokine inhibition [13,14].

Sustained inflammatory activity may eventually trigger pericardial remodeling. Cytokine-driven fibroblast activation promotes extracellular matrix deposition and a progressive loss of compliance. Over time, this process can result in pericardial thickening and calcification, with reversibility depending on the balance between active inflammation and fibrosis [7,17].

### 3.3 From Inflammation to Constriction: A Pathophysiologic Continuum

Pericardial disease progresses along a continuum rather than in discrete stages. Early in the disease course, inflammation predominates, and pericardial compliance may be preserved despite the presence of edema or effusion. At this stage, the condition is often reversible with appropriate anti-inflammatory therapy [7,17].

Fibrotic remodeling develops with persistent or recurrent inflammation. Pericardial stiffness increases, and early constrictive physiology may emerge. This intermediate phase, often referred to as inflammatory or transient constriction, is clinically important because aggressive medical therapy may still reverse the process [7,17].

Chronic constrictive pericarditis develops in patients with advanced disease. Dense fibrosis and calcification severely restrict diastolic filling, exaggerate ventricular interdependence, elevate systemic venous pressure, and reduce cardiac output. At this stage, medical therapy is usually ineffective, and pericardiectomy is often necessary for definitive management [7,12,17].

### 3.4 Imaging–Pathology Correlation

Imaging plays a central role in linking pericardial pathology to the clinical presentation. Echocardiography remains essential for functional assessment, including determining ventricular interdependence and respiratory variation in filling patterns [17,30]. However, its ability to characterize tissue composition is limited.

Cardiac CT accurately defines the pericardial anatomy and detects calcification, a hallmark of chronic constriction [12]. CMR imaging provides unique tissue characterization; T2-weighted imaging identifies pericardial edema, whereas late gadolinium enhancement (LGE) reflects active inflammation or fibrosis [18,19,20,33,34].

Importantly, CMR can distinguish between reversible inflammatory diseases and irreversible fibrotic constrictions. This differentiation directly informs therapeutic decisions, guiding the selection between medical therapy and surgical intervention [18,19,20,33,34]. In selected cases, PET may provide complementary information regarding metabolic inflammatory activity, particularly when conventional imaging findings are inconclusive or discordant [20].

In summary, normal pericardial function depends on ventricular coupling and restraint. In contrast, pericardial disease is primarily driven by innate immune–mediated inflammation, with IL-1 playing a central role. The transition from acute pericardial inflammation to chronic constrictive physiology occurs along a continuum shaped by the duration and intensity of inflammatory activity. Recent advances in multimodality imaging have enabled more precise phenotyping of pericardial disease, linking the underlying pathophysiology with clinical decision-making and supporting the development of personalized, mechanism-based therapeutic strategies.

## 4. Clinical Classification and Spectrum of Pericardial Syndromes

Pericardial diseases comprise a heterogeneous group of clinical syndromes with distinct presentations, pathophysiologies, and prognostic implications. Accurate clinical classification is essential for diagnosis and therapeutic decision-making. Contemporary concepts emphasize that these syndromes represent a dynamic spectrum rather than isolated entities shaped by inflammatory activity, structural remodeling, or hemodynamic consequences [3].

### 4.1 Definitions of Pericardial Syndromes

Acute pericarditis is defined as pericardial inflammation with a symptom duration of less than 4–6 weeks. The typical features include pleuritic chest pain, characteristic electrocardiographic changes, and variable pericardial effusion. Most cases respond to anti-inflammatory therapy; however, inadequate control of inflammation may lead to disease progression [6,22,35].

Incessant pericarditis refers to persistent symptoms lasting longer than 4–6 weeks without a symptom-free interval, despite ongoing therapy. It reflects sustained inflammatory activity and occupies an intermediate position between acute and recurrent disease, often prompting reassessment of etiology and treatment strategy [3,4,5,22].

Recurrent pericarditis is characterized by symptom recurrence after a symptom-free interval of at least 4–6 weeks following the resolution of an initial episode. It is associated with repeated inflammatory flares, cumulative morbidity, corticosteroid dependence, and impaired quality of life [3,4,5,6,11,13]. Increasing evidence indicates that recurrent pericarditis is frequently an autoinflammatory condition rather than repeated episodes of *de novo* acute inflammation [11,13,14].

Chronic pericardial disease is a pericardial pathology persisting beyond 3 months and is commonly associated with structural remodeling, including fibrosis and calcification. Clinical manifestations vary and may include persistent effusion, recurrent inflammation, or constrictive physiology [12,17].

Together, these definitions illustrate the temporal progression of pericardial disease and reinforce the importance of early, mechanism-based intervention [3,4,5,22].

### 4.2 Pericardial Effusion, Cardiac Tamponade, and Constrictive Pericarditis

Pericardial effusion, cardiac tamponade, and constrictive pericarditis represent distinct but interrelated manifestations of pericardial disease, each defined by specific pathophysiologic and clinical features [12,36,37].

Pericardial effusion results from an imbalance between the production of pericardial fluid and resorption and may occur in both inflammatory and non-inflammatory conditions. The clinical impact depends primarily on the rate of fluid accumulation rather than on the absolute volume. Slowly accumulating effusions may remain asymptomatic, whereas rapid accumulation can cause hemodynamic compromise even with small volumes [36,38].

Cardiac tamponade is the most severe hemodynamic consequence of pericardial effusion. Elevated intrapericardial pressure impairs diastolic filling and reduces the stroke volume and cardiac output. Hypotension, tachycardia, and systemic venous congestion are the typical features. Importantly, tamponade is a functional diagnosis defined by hemodynamic effects rather than effusion size alone [38,39,40].

Constrictive pericarditis results from chronic pericardial fibrosis and a loss of compliance. Restricted diastolic filling results in exaggerated ventricular interdependence and dissociation between intrathoracic and intracardiac pressures [12,37]. Clinically, patients present with signs of right-sided heart failure, including peripheral edema, ascites, and hepatic congestion [37].

These syndromes have various underlying mechanisms of action. Effusion and tamponade are primarily fluid-mediated, whereas constriction is driven by structural remodeling [36]. Effusive constrictive pericarditis represents a hybrid phenotype in which the constrictive physiology persists despite the drainage of pericardial effusion, highlighting the coexistence of inflammatory and fibrotic processes [36,41].

Beyond the diagnosis, interventional management is mandatory for patients with hemodynamically significant effusions or cardiac tamponade. Pericardiocentesis remains the gold standard for immediate hemodynamic stabilization and is typically performed under real-time echocardiographic or fluoroscopic guidance to minimize procedural complications. In patients presenting recurrent malignant effusions or loculated collections, or when a pericardial tissue biopsy is required for definitive diagnostic evaluation, surgical drainage via a subxiphoid pericardial window or pericardiotomy may be preferred. A multidisciplinary approach that integrates clinical urgency and underlying etiology is essential to determine the optimal timing and modality of intervention.

### 4.3 Clinical Implications

Understanding the spectrum of pericardial syndrome has important clinical implications for patient management. Early identification of patients at increased risk of recurrence or disease progression enables timely escalation of anti-inflammatory therapy while minimizing unnecessary or inappropriate corticosteroid exposure. In particular, distinguishing reversible inflammatory constriction from irreversible fibrotic disease is critical because this distinction directly informs the choice between continued medical therapy and referral for surgical pericardiectomy.

Overall, clinical classification provides a coherent framework that integrates symptom duration, underlying pathophysiology, and therapeutic decision-making, underscoring the importance of individualized mechanism-based management across the full spectrum of pericardial disease.

## 5. Acute Pericarditis: Diagnosis, Risk Stratification, and Initial Management

Acute pericarditis is the most common clinical manifestation of pericardial disease and a frequent cause of nonischemic chest pain. Although many cases are self-limiting, early diagnostic accuracy and risk-based management are critical for preventing complications and recurrence. Contemporary practice emphasizes a structured approach integrating clinical criteria, electrocardiography, biomarkers, and imaging, followed by early risk stratification [3,4,5,9,22].

### 5.1 Diagnostic Criteria and Supportive Findings

Acute pericarditis is diagnosed based on the presence of at least two of four core criteria: typical pericarditic chest pain, pericardial friction rub, characteristic electrocardiographic changes, and new or worsening pericardial effusion [9,22]. These criteria support bedside diagnosis but are complemented by supportive findings that improve diagnostic confidence and prognostic assessment.

Supportive features include elevated inflammatory markers, leukocytosis, and imaging evidence of pericardial inflammation [9,22]. Transthoracic echocardiography is recommended at the initial evaluation to assess effusion and hemodynamic impact [3,4,5,42]. In selected cases, CMR imaging can provide evidence of pericardial edema or LGE, supporting active inflammation and identifying concomitant myocardial involvement [33,43]. Diagnostic evaluation should also consider secondary causes when suggested by clinical context, such as systemic autoimmune disease, tuberculosis, malignancy, or drug-related injury [3,4,5,9].

### 5.2 Electrocardiogram (ECG) Patterns and Key Differential Diagnosis

Electrocardiography remains the cornerstone in the diagnosis of acute pericarditis, with characteristic findings that evolve over time. The classic ECG pattern progresses through four stages, although not all patients exhibit all stages or progress through them sequentially.

Stage I, the most distinctive phase, typically shows diffuse, concave ST-segment elevation with PR-segment depression, which is often most evident in the limb leads, reflecting epicardial and atrial involvement. In Stage II, ST and PR abnormalities resolve toward baseline. Stage III is characterized by T-wave inversion after the normalization of ST segments, and Stage IV represents the resolution of T-wave changes, although mild non-specific abnormalities may persist in some patients [35,44].

As ECG findings strongly influence acute management decisions, accurately differentiating pericarditis from other urgent causes of chest pain is essential. ST-elevation myocardial infarction (STEMI) typically exhibits regional ST-segment elevation, reciprocal ST depression in opposing leads, and dynamic changes consistent with the coronary territory. In contrast, acute pericarditis usually presents with diffuse ST-segment elevation with no reciprocal depression, except in the aVR and V1, and is often accompanied by PR-segment depression [44]. The clinical context remains crucial: pleuritic chest pain relieved by leaning forward and a pericardial friction rub favor pericarditis, whereas ischemic chest pain and high-risk coronary features should prompt an emergent coronary evaluation [3,4,5,35,44].

Another important differential diagnosis is myocarditis or myopericarditis, in which myocardial injury results in elevated troponin levels and may be accompanied by arrhythmias or ventricular dysfunction. In myopericarditis, typical pericarditic symptoms are present along with biochemical evidence of myocardial injury, whereas in perimyocarditis, myocardial involvement predominates over the pericardial features [43]. In cases with suspected myocardial involvement, CMR imaging is particularly valuable for risk stratification and for guiding decision-making relative to imposing activity restriction and follow-up [19,33,34,43].

### 5.3 Risk Stratification and Site-of-Care Decisions

Early risk stratification is a central component of acute pericarditis management, helping identify patients who require hospitalization, etiologic evaluation, and stringent monitoring. High-risk features are associated with an increased likelihood of complications, including large pericardial effusion, cardiac tamponade, treatment failure, and secondary etiologies requiring specific therapy [3,4,5,9].

Clinical decision-making can be guided by two key considerations: the risk of imminent hemodynamic compromise or early treatment failure and the probability of a non-idiopathic cause warranting targeted diagnostic evaluation. Patients with high-risk features should be managed as inpatients through serial assessments and extended workups. In contrast, low-risk patients with typical clinical features and a favorable early response to therapy may be treated in an outpatient setting with close follow-up and inflammatory marker–guided treatment adjustment.

Overall, a structured site-of-care strategy balances patient safety with resource utilization and reduces unnecessary admissions, while minimizing the risk of missed complications or occult secondary causes [3,4,5,9,22].

### 5.4 Initial Therapy and Common Pitfalls

First-line therapy for acute pericarditis consists of high-dose aspirin or NSAIDs in combination with colchicine, provided there are no contraindications [3,4,5,9,10,22,25]. This approach aims to achieve rapid symptom control and complete suppression of pericardial inflammation during the initial episode, thereby preventing incomplete resolution, which may predispose patients to subsequent disease chronicity.

NSAIDs should be administered at full anti-inflammatory doses rather than simple analgesic doses. Common regimens include 600–800 mg ibuprofen every 8 h, 25–50 mg indomethacin every 8 h, and 750–1000 mg aspirin every 8 h. Aspirin is preferred in patients with ischemic heart disease or post–myocardial infarction pericarditis [1,3,4,5,9,22]. Therefore, gastroprotection using proton pump inhibitors is recommended. The duration of NSAID therapy should not be predefined but should instead be guided by the complete resolution of symptoms and normalization of inflammatory markers, particularly C-reactive protein (CRP), which should be monitored serially. Premature tapering or discontinuation before full inflammatory control remains a frequent and avoidable cause of treatment failure [9,10,22,25].

Colchicine should be initiated early as an adjunct to NSAIDs in most patients with acute pericarditis. Randomized controlled trials have demonstrated that colchicine reduces symptom persistence and lowers the risk of recurrence after an initial episode [45,46]. Weight-adjusted dosing is recommended (0.5–0.6 mg once daily for patients <70 kg and twice daily for those ≥70 kg), and therapy should generally be continued for approximately 3 months in acute pericarditis [9,10,22]. Dose adjustment is required in older patients, in those with renal dysfunction, or in the presence of relevant drug interactions. Mild gastrointestinal intolerance is common but can usually be managed with dose reduction rather than with discontinuation [9,10,47].

One of the most important therapeutic pitfalls of acute pericarditis is the early or indiscriminate use of corticosteroids. Although corticosteroids often provide rapid symptom relief, their early use, particularly at high doses, has been consistently associated with higher recurrence rates, prolonged disease course, and corticosteroid dependence [25,47]. Therefore, corticosteroids should be avoided as a first-line therapy and reserved for specific clinical scenarios, including pericarditis associated with systemic autoimmune disease, contraindications to NSAIDs and colchicine, and truly refractory cases [3,4,5,10,25]. When corticosteroids are necessary, the lowest effective dose should be used with very slow tapering guided by sustained clinical remission and normalization of inflammatory markers.

Supportive non-pharmacological measures play an important role in initial management. Temporary restriction of strenuous physical activity is recommended until symptom resolution and normalization of inflammatory markers, particularly in physically active individuals and athletes [3,9]. Patient education regarding the inflammatory nature of the disease, the importance of adherence to therapy despite early symptom improvement, and early recognition of warning symptoms is essential. Failure to adequately address these aspects represents an underappreciated contributor to incomplete inflammatory control and disease relapse [9,25].

## 6. Recurrent Pericarditis and the Autoinflammatory Paradigm

Recurrent pericarditis is one of the most challenging conditions within the spectrum of pericardial diseases. A substantial proportion of patients experience recurrent inflammatory flares associated with prolonged treatment, frequent relapses, and an impaired quality of life. Increasing evidence indicates that recurrent pericarditis is a distinct clinical entity driven by autoinflammatory mechanisms rather than by repeated episodes of acute inflammation [13,32,48].

### 6.1 Definition and Clinical Burden

Recurrent pericarditis is defined as symptom recurrence after a symptom-free interval of at least 4–6 weeks following resolution of the initial episode [3,4,5,22]. Recurrences typically present with chest pain and elevated inflammatory markers, whereas effusion is less frequent than during the initial episode [13,48].

The clinical burden extends beyond episodic pain. Recurrent hospitalization, prolonged anti-inflammatory therapy, and activity restriction contribute to an impaired quality of life [48]. Steroid dependence is a common clinical challenge in patients with recurrent pericarditis. Although corticosteroids provide rapid symptom relief, dose tapering often precipitates relapse and is associated with cumulative adverse effects, underscoring the need for steroid-sparing therapies [11,47]. Recurrent inflammation may also contribute to long-term pericardial remodeling. Although progression to constrictive pericarditis is uncommon, recurrent pericarditis should be viewed as a chronic inflammatory condition requiring a long-term disease-modifying management approach [49].

### 6.2 Autoinflammatory Mechanism in Recurrent Pericarditis

Current evidence supports an autoinflammatory mechanism driven predominantly by innate immune activation [13,32]. IL-1 plays a central role by promoting leukocyte recruitment and sustained pericardial inflammation [14,32]. Dysregulated IL-1 signaling can lead to recurrent inflammatory episodes, even in the absence of an identifiable trigger. Beyond dysregulated innate signaling, recent evidence has further elucidated the complexity of IL-1 counter-regulation. Emerging data suggest that the presence of anti-interleukin-1 receptor antagonist (IL-1Ra) autoantibodies in a subset of patients with idiopathic recurrent pericarditis indicates that impaired endogenous inhibition of IL-1 contributes to a sustained inflammatory state [50].

This autoinflammatory paradigm explains several characteristic features of recurrent pericarditis. In many patients, extensive evaluation fails to identify the underlying infectious or autoimmune causes. Conventional immunosuppressive therapies targeting adaptive immunity often exhibit limited efficacy, whereas IL-1 inhibition has demonstrated marked clinical benefits in patients with refractory or steroid-dependent disease [13,14,15]. Consistent with this model, most patients lack disease-specific autoantibodies, and histopathological findings do not typically reflect a predominant adaptive immune activation [11,13].

### 6.3 Limitations of Conventional Treatments

Conventional therapies for recurrent pericarditis include colchicine, corticosteroids, and, in selected cases, non-specific immunosuppressive agents. Although these treatments remain the mainstay of management, their significant limitations contribute to incomplete disease control in a subset of patients.

Colchicine remains the cornerstone of recurrence prevention and significantly reduces the risk of relapse [45,46]. However, colchicine does not fully prevent recurrence in all patients, particularly in those with frequent flares or with steroid dependence, and its use may be limited by gastrointestinal intolerance or comorbid renal or hepatic dysfunction [48].

Corticosteroids are effective for acute symptom relief but are associated with higher recurrence rates and cumulative toxicity when used as long-term therapy [11,13,47]. Repeated tapering often precipitates relapse, leading to steroid dependence and limiting its use as a durable treatment option.

Non-specific immunosuppressive agents, such as azathioprine or methotrexate, may be considered in selected cases, particularly in patients with systemic autoimmune diseases. However, their delayed onset of action and limited efficacy in idiopathic recurrent pericarditis limit their routine use [9,13].

Overall, these limitations highlight the need for targeted and durable therapeutic strategies for patients with recurrent pericarditis.

### 6.4 Transition to Targeted Therapy

Recognition of recurrent pericarditis as an autoinflammatory disease has transformed management strategies. The identification of IL-1 as a key mediator has enabled targeted therapies that achieve rapid symptom control, sustained remission, and steroid-sparing effects [14,15,21]. These advances have underpinned the expanding role of IL-1 inhibitors, as discussed in the following section.

## 7. Targeted Anti-Inflammatory Therapy: IL-1 Inhibitors

### 7.1 Rationale for IL-1 Blockade

The conceptualization of recurrent pericarditis as an autoinflammatory disorder has reshaped therapeutic priorities from broad anti-inflammatory suppression toward pathway-specific interventions [11,13]. A central feature of autoinflammatory pericarditis is dysregulated innate immune activation, with IL-1 acting as an upstream amplifier of the inflammatory cascade [13,32]. IL-1α and IL-1β promote endothelial activation, leukocyte recruitment, and sustained cytokine signaling that perpetuate pericardial pain, systemic inflammatory markers such as CRP, and recurrent effusions [11,13,32]. Notably, recent evidence has further elucidated the complexity of IL-1 counter-regulation, with emerging data suggesting the presence of anti-IL-1Ra autoantibodies in patients with idiopathic recurrent pericarditis. This suggests that impaired endogenous inhibition of IL-1 contributes to a sustained inflammatory state [50]. This biological framework provides a strong mechanistic rationale for IL-1 blockade as a disease-modifying strategy, rather than symptomatic suppression alone. Recent reviews and expert guidance emphasize that IL-1 inhibition is particularly relevant in patients with an “inflammatory phenotype” of recurrent pericarditis, often characterized by active symptoms, elevated CRP, and recurrent flares during glucocorticoid tapering, where conventional therapy frequently fails to achieve durable, steroid-free remission [9,11,14,25].

### 7.2 Anakinra

Anakinra is a recombinant IL-1Ra that competitively inhibits IL-1α and IL-1β signaling at the IL-1 type I receptor, thereby interrupting upstream innate immune activation central to autoinflammatory pericarditis [14,32]. Pivotal evidence from randomized trials supporting the efficacy of anakinra therapy in recurrent pericarditis was provided by the AIRTRIP trial (JAMA, 2016) [21]. The study enrolled patients with recurrent pericarditis who were resistant to colchicine, dependent on corticosteroids, and had elevated CRP levels. Anakinra (2 mg/kg/day, maximum 100 mg) significantly reduced the recurrence risk compared with placebo during the randomized withdrawal phase, demonstrating effective control of inflammation and prevention of relapse in a high-risk population [21]. A key clinical feature of anakinra is its rapid onset of action, with prompt improvement in chest pain and normalization of inflammatory markers, particularly in patients with an inflammatory phenotype [9,11]. In practice, anakinra is commonly used as a steroid-sparing strategy in patients with corticosteroid-dependent recurrent pericarditis, as it allows gradual tapering of glucocorticoids once symptom control and CRP normalization are achieved [9,25]. Abrupt discontinuation may precipitate relapse, and tapering strategies are typically individualized based on disease chronicity, relapse history, and biomarker trends [9,11]. Injection-site reactions are the most commonly reported adverse events. Furthermore, infection risk requires routine clinical monitoring, and baseline screening for active infection is generally recommended prior to initiation, consistent with the broader experience with inflammatory diseases [14,25].

### 7.3 Rilonacept

Rilonacept is a soluble decoy receptor (“IL-1 trap”) that binds IL-1α and IL-1β, thereby attenuating downstream inflammatory signaling [14,16]. The phase 3 RHAPSODY trial provided high-quality randomized evidence demonstrating the rapid resolution of recurrent pericarditis episodes and a significantly lower risk of recurrence compared with placebo [15]. Importantly, the trial incorporated a run-in phase during which background therapies, including glucocorticoids and colchicine, could be tapered to reflect real-world treatment objectives [15].

One major clinical advantage of rilonacept is its potential for robust steroid-sparing effects. In the RHAPSODY trial, many patients transitioned to rilonacept monotherapy after stabilization, supporting a pragmatic approach of initiating IL-1 blockade, achieving inflammatory control, and then subsequently tapering or discontinuing glucocorticoids and other anti-inflammatory agents as clinically appropriate [15,25].

For recurrent pericarditis, rilonacept is administered at a 320 mg loading dose followed by 160 mg subcutaneously once weekly, according to the approved prescribing information [51]. Compared with the daily injections required for anakinra, this once-weekly regimen may improve adherence and long-term treatment acceptability [16]. Rilonacept shares infection-related considerations typical of immunomodulatory therapy. Laboratory changes, including higher lipid levels, have been reported, and periodic laboratory monitoring is recommended during therapy [16].

### 7.4 Guidelines and Expert Consensus: Practical Implementation

Recent clinical guidelines support the integration of IL-1 inhibitors into stepwise management algorithms for recurrent pericarditis, particularly in patients with objective evidence of inflammation, colchicine resistance, or corticosteroid dependence. The 2025 American College of Cardiology (ACC) Concise Clinical Guidelines emphasize minimizing inappropriate corticosteroid exposure and incorporating IL-1 inhibition in recurrent and refractory disease when inflammatory activity is documented [4]. In parallel, the 2025 European Society of Cardiology (ESC) guidelines for inflammatory myocardial and pericardial syndromes highlight phenotype-driven management strategies and support targeted IL-1 blockade in patients with demonstrable inflammatory diseases, guided by clinical presentation, biomarkers, and imaging findings [5].

#### Practical Clinical Strategy

(1) Confirm inflammatory phenotype: recurrent symptoms with elevated CRP and/or imaging evidence excluding secondary causes such as infection or malignancy based on the clinical context [3,4,5,9,11,23,25].

(2) Optimize conventional therapy: ensure adequate NSAID/aspirin and colchicine use with inflammation-guided tapering [9,22,25].

(3) Identify potential candidates for escalation: frequent recurrence, corticosteroid dependence, or colchicine resistance, all of which support IL-1 inhibitor initiation [9,11,15,21].

(4) Implement steroid-sparing transitions: taper glucocorticoids gradually once IL-1 blockade achieves control of symptoms and inflammatory markers [15,16,25].

(5) Monitor safety and individualize duration:treatment duration and tapering should be guided by relapse history and inflammatory activity rather than by fixed timelines [4,5,9,11].

## 8. Multimodality Imaging in Pericardial Diseases

Multimodal imaging has become a cornerstone of contemporary pericardial disease management, extending beyond the simple detection of pericardial effusion. Modern imaging enables the integrated assessment of pericardial anatomy, hemodynamic consequences, and inflammatory activity, thereby facilitating diagnosis, risk stratification, therapeutic decision-making, and longitudinal follow-up. An approach that leverages the complementary strengths of echocardiography, cardiac CT, CMR, and 18F-FDG-PET is now widely endorsed in expert consensus and guideline-based care (Table 1) [3,4,5,18,19,20].

**Table 1.** **Comparison of multimodality imaging in the diagnosis and management of pericardial diseases**.

Modality	Key strengths	Main limitations	Clinical role in pericardial disease
Echocardiography	Real-time, portable, bedside assessment; excellent for hemodynamics.	Limited acoustic windows; poor tissue characterization.	First-line tool for diagnosing effusion and cardiac tamponade.
Cardiac computed tomography	High-resolution for calcification; fast acquisition; detailed anatomy.	Ionizing radiation; limited functional/tissue information.	Gold standard for detecting pericardial calcification; surgical planning.
Cardiac magnetic resonance imaging	Superior tissue characterization (edema/LGE); functional assessment.	High cost; long scan time; contraindicated with certain implants.	Allows distinguishing active inflammation from irreversible fibrosis.
18F-fluorodeoxyglucose positron emission tomography	High sensitivity for metabolic activity; identifies systemic disease.	Radiation; physiologic myocardial uptake; requires specific dietary prep.	Identifying metabolic inflammatory activity in refractory or malignant cases.

LGE, late gadolinium enhancement.

### 8.1 Echocardiography

Transthoracic echocardiography remains the first-line imaging modality for suspected pericardial disease, owing to its availability, portability, and capacity for real-time hemodynamic assessment. In acute settings, it is essential to detect pericardial effusion and evaluate its physiological significance.

Beyond estimating effusion size and distribution, echocardiography focuses on identifying the features of hemodynamic compromise. In cardiac tamponade, characteristic findings include right atrial systolic collapse, right ventricular diastolic collapse, exaggerated respiratory variation in transvalvular Doppler flows, and inferior vena cava plethora, which underscore that tamponade is a functional diagnosis driven by pressure–volume interactions rather than effusion size alone [38,39,40].

Echocardiography is also central to the evaluation of constrictive pericarditis, for which functional assessment is predominant. The key features include ventricular interdependence, septal bounce, a dissociation between intrathoracic and intracardiac pressures, and marked respiratory variation in mitral and tricuspid inflow velocities. Tissue Doppler imaging demonstrating preserved or increased early diastolic mitral annular velocity (annulus paradoxus) aids in distinguishing constrictive pericarditis from restrictive cardiomyopathy (RCM) [37,41]. However, echocardiography has a limited ability to directly assess pericardial thickness, calcification, and inflammatory activity, necessitating complementary imaging modalities.

### 8.2 Cardiac Computed Tomography

Cardiac CT provides a high-resolution anatomical visualization of the pericardium and is particularly valuable for assessing pericardial thickness and calcification. Cardiac CT is the imaging modality of choice when calcific constriction is suspected, as even minimal pericardial calcium can be detected with high sensitivity [18,34].

In patients with suspected constrictive pericarditis, CT delineates the extent and distribution of pericardial thickening and clarifies the relationship between the pericardium and the adjacent cardiac structures. These anatomical details are especially relevant for the preoperative planning of pericardiectomy, where calcification burden and distribution influence surgical complexity and risk [12,18]. CT can also characterize pericardial effusions based on attenuation values and identify associated mediastinal or pulmonary abnormalities suggestive of secondary etiologies, such as malignancy or infection. Nonetheless, CT provides limited information on active inflammation and lacks the functional and tissue characterization capabilities of echocardiography and CMR imaging.

### 8.3 Cardiac Magnetic Resonance Imaging

CMR imaging has emerged as the central modality for pericardial tissue characterization, uniquely enabling the assessment of inflammatory activity, edema, and fibrosis. This capability has transformed the clinical approach to pericardial disease, particularly for distinguishing potentially reversible inflammatory states and irreversible fibrotic constriction [18,19,20].

T2-weighted imaging allows the detection of pericardial edema, reflecting active inflammation, whereas LGE identifies increased extracellular space and vascularity within the pericardium. Taken together, these findings support an inflammatory phenotype and suggest potential responsiveness to anti-inflammatory or targeted therapies. Conversely, the absence of edema with dense pericardial thickening or calcification favors chronic fibrotic constriction, in which surgical pericardiectomy is more likely to be required [33,34].

In addition to tissue characterization, CMR imaging provides a comprehensive functional assessment of ventricular volume, septal motion, and ventricular interdependence and can detect concomitant myocardial involvement with important prognostic and management implications.

### 8.4 Adjunctive Role of FDG-PET Imaging

FDG-PET imaging, typically combined with CT imaging, may provide complementary information in selected patients by identifying metabolically active pericardial inflammation. Increased pericardial FDG uptake has been described in inflammatory, infectious (including tuberculous), and malignant pericardial diseases and may aid etiologic evaluation when conventional imaging findings are inconclusive [23,28,29].

In constrictive pericarditis, focal or diffuse FDG uptake suggests an active inflammatory phenotype with potential reversibility, supporting a trial of anti-inflammatory or immunomodulatory therapy, whereas the absence of uptake is indicative of chronic fibrotic diseases. However, the routine use of PET is limited by physiological myocardial uptake, the need for a strict patient preparation, limited spatial resolution for thin pericardial structures, radiation exposure, and a lack of standardized interpretation criteria. Accordingly, the current expert consensus considers PET as an adjunctive modality reserved for selected clinical scenarios rather than as a first-line imaging tool [4,5,20].

### 8.5 Integrating Multimodality Imaging Into Clinical Decision-Making

The value of multimodal imaging lies in its integration with phenotype-driven management strategies. Echocardiography establishes functional diagnosis and hemodynamic risk; CT defines anatomical features relevant to chronicity and surgical planning, while CMR bridges anatomy and biology by identifying inflammatory activity and tissue composition.

In patients with recurrent pericarditis, imaging complements clinical and biomarker data to identify patients with active inflammation who may benefit from targeted therapies, including IL-1 inhibition. In constrictive disease, imaging is central to distinguishing transient inflammatory constriction from fixed fibrotic disease, directly informing the choice between medical therapy and surgical intervention [4,5,9,10,33,34].

## 9. Constrictive Pericarditis and Surgical Considerations

Constrictive pericarditis represents the advanced end of the pericardial disease spectrum and is characterized by a rigid, noncompliant pericardium that impairs diastolic filling [24,41]. Although relatively uncommon, constrictive pericarditis is associated with substantial morbidity and poses critical diagnostic and therapeutic challenges, particularly in the differential diagnosis with myocardial restrictive processes and in determining the optimal timing of surgical intervention [7,8,17,37].

### 9.1 Diagnosis and Differential Diagnosis

The diagnosis of constrictive pericarditis requires the integration of clinical presentation, multimodal imaging, and invasive hemodynamic assessment in selected cases [17,37,49]. Patients typically present with manifestations of right-sided heart failure, including peripheral edema, ascites, hepatic congestion, and exertional dyspnea, often in the absence of significant left ventricular systolic dysfunction [39,41].

A central diagnostic challenge is distinguishing constrictive pericarditis from RCM, as both conditions share overlapping clinical features [17,37]. Although the underlying pathophysiologies are fundamentally different, in constrictive pericarditis, diastolic filling is limited by an external noncompliant pericardium despite relatively preserved myocardial compliance, whereas RCM is characterized by intrinsic myocardial stiffness [31,41].

Multimodal imaging plays a pivotal role in this distinction. Echocardiography may demonstrate ventricular interdependence, septal bounce, and exaggerated respiratory variation in transvalvular inflow velocities in constrictive pericarditis, which are findings that are typically absent or attenuated in RCM [37,41,49]. Tissue Doppler imaging demonstrating preserved or increased early diastolic mitral annular velocity (annulus paradoxus) further supports constriction [37,41]. Cardiac CT can identify pericardial thickening and calcification [18,34], whereas CMR imaging provides tissue characterization and functional assessment that contribute to clarifying the relative contributions of pericardial versus myocardial pathology [19,20].

When noninvasive evaluation remains inconclusive, an invasive hemodynamic assessment may demonstrate findings characteristic of constriction, including equalization of diastolic pressure and discordant ventricular pressure changes with respiration [36,37]. Accurate differentiation is essential, as management strategies and prognoses differ markedly between constrictive pericarditis and RCM [17,37,49].

### 9.2 Medical Versus Surgical Decision-Making

A critical step in managing constrictive pericarditis is determining whether the disease represents a potentially reversible inflammatory process or a fixed fibrotic constriction requiring surgical intervention [17,37]. This distinction has gained increasing importance with advances in multimodal imaging and anti-inflammatory therapy [33,34].

Inflammatory constriction refers to a potentially reversible phase characterized by active pericardial inflammation with preserved or partially preserved pericardial elasticity. Imaging findings, including pericardial edema and LGE, support this diagnosis [33,34]. In selected patients, aggressive anti-inflammatory therapy, including NSAIDs, colchicine, and, in refractory inflammatory phenotypes, targeted anti-inflammatory agents such as IL-1 inhibition, may lead to symptomatic improvement and resolution of constrictive physiology [9,11,14,21,25]. Strict monitoring with serial imaging and inflammatory biomarkers is essential during medical management to assess treatment response and to guide escalation or de-escalation of therapy [33,34].

In contrast, chronic fibrotic constriction is characterized by a dense pericardial fibrosis, which is frequently accompanied by calcification, with little or no evidence of active inflammation [7,8,24]. In such cases, medical therapy is unlikely to restore pericardial compliance, and surgical pericardiectomy remains the definitive treatment [7,8,12]. When performed in appropriately selected patients, pericardiectomy can result in substantial symptomatic and hemodynamic improvement; however, it carries nontrivial perioperative risks, particularly in patients presenting advanced disease, extensive calcification, or significant comorbidities [7,8,12,37].

Indications for pericardiectomy generally include persistent constrictive physiology with refractory symptoms despite optimized medical therapy, imaging evidence of chronic fibrotic disease, and acceptable surgical risk [12,17,37,49]. Timing of intervention is critical, as delayed referral may result in progression toward irreversible myocardial involvement or end-organ dysfunction, adversely affecting surgical outcomes [7,8,12,37]. Accordingly, early recognition and timely multidisciplinary evaluation are central to optimizing outcomes in patients presenting constrictive pericarditis [12,17,37,49].

## 10. Prognosis, Follow-Up, and Future Directions

The prognosis of pericardial disease varies widely, depending on the etiology, clinical phenotype, and response to therapy [1,25]. Most patients with idiopathic or viral acute pericarditis recover completely with appropriate treatment [3,4,5,22]. In contrast, patients with recurrent and constrictive pericarditis are associated with higher morbidity and, in selected cases, persistent functional limitation and impaired quality of life [11,37,48].

In recurrent pericarditis, prognosis is influenced by relapse frequency, the degree of inflammatory activity, and cumulative exposure to corticosteroids [9,11,47]. The introduction of targeted anti-inflammatory therapies has favorably changed outcomes in refractory disease [14,15,21]. These agents enable a durable remission and facilitate steroid-sparing management [15,16,48]. Accordingly, long-term follow-up should address not only control of symptoms but also of treatment-related toxicity and structural progression [9,25].

Imaging-guided follow-up is now central to contemporary pericardial disease management [3,4,5,18,19,20]. Serial echocardiography allows the monitoring of effusion dynamics and hemodynamic consequences [37,40]. CMR imaging provides an objective assessment of residual inflammation and pericardial remodeling [19,20,33,34]. Furthermore, an inflammation-guided strategy supports individualized treatment duration and tapering and reduces the risk of premature withdrawal and relapse [9,25].

Pericardial disease management is moving toward a precision medicine paradigm [4,5,20]. Clinical phenotypes, biomarkers, and multimodal imaging are becoming increasingly integrated to guide therapy [5,9,25]. Ongoing research aims to refine the risk stratification and optimize the sequencing and duration of targeted treatments [12,14,46]. Beyond conventional inflammatory markers, such as CRP, the erythrocyte sedimentation rate (ESR), and troponin levels, the evolving landscape of biomarkers offers promising utility for precision management. Recent evidence has highlighted the diagnostic and prognostic potential of emerging biomarkers, including various ILs, soluble urokinase plasminogen activator receptors (suPAR), and microRNAs, which may further refine inflammation-guided therapy and longitudinal monitoring. Ultimately, improving the prediction of treatment response and distinguishing reversible inflammatory phenotypes from irreversible disease and remain key goal for future investigation [5,33,34,52].

## 11. Conclusions

Pericardial diseases encompass a broad and dynamic spectrum of clinical syndromes that extend beyond the traditional perspective of acute self-limited inflammation. Contemporary evidence supports the understanding of pericardial disease as a continuum, ranging from acute pericarditis to recurrent inflammatory states, and in selected cases, chronic constrictive physiology. Advances in multimodal imaging technologies have substantially improved diagnostic accuracy and risk stratification. Echocardiography, cardiac CT, and CMR imaging allow a detailed assessment of the pericardial anatomy, hemodynamic consequences, and inflammatory activity. In parallel, improved immunopathological insight, particularly the recognition of recurrent pericarditis as an autoinflammatory condition, has enabled the development of targeted therapies that address the underlying disease mechanisms rather than providing only symptomatic relief. From a clinical standpoint, a stepwise phenotype-driven approach is essential. Early risk stratification, appropriate first-line therapy, avoidance of unnecessary corticosteroid exposure, and timely escalation of targeted anti-inflammatory treatment are central to optimizing patient outcomes. In constrictive pericarditis, careful differentiation between inflammatory and fibrotic phenotypes is critical. This distinction guides the choice between medical therapy and surgical intervention and highlights the importance of multidisciplinary evaluation. In conclusion, the continued integration of imaging, biomarkers, and targeted therapies is expected to further refine pericardial disease management. A modern precision-based framework offers the potential to improve long-term outcomes, reduce treatment-related morbidity, and enhance the quality of life across the full spectrum of pericardial diseases.
